# Lysine deficiency within a conserved lysine desert is critical for EEL-1/HUWE1 to support ubiquitin proteasome system function

**DOI:** 10.1371/journal.pgen.1012273

**Published:** 2026-08-10

**Authors:** Katherine S. Yanagi, Brenda J. Chen, Sheikh Omar Kunjo, Irini Topalidou, Nicolas Lehrbach

**Affiliations:** Basic Sciences Division, Fred Hutchinson Cancer Center, Seattle, Washington, United States of America; University of Wisconsin-Madison, UNITED STATES OF AMERICA

## Abstract

The ubiquitin proteasome system (UPS) is the primary mechanism for targeted protein degradation in eukaryotic cells. Dysfunction of this system is a driver of human disease and a hallmark of aging and late-onset neurodegenerative disorders. Understanding the mechanisms that ensure robust protein turnover may provide new avenues for treatment in these contexts. E3 ubiquitin ligases play critical roles in supplying ubiquitinated substrates to the proteasome, with HUWE1 being an enormous, versatile, and highly conserved member of this family. Here, we show that the *C. elegans* HUWE1 ortholog EEL-1 contributes to robust protein turnover, particularly during conditions that challenge the proteolytic capacity of the proteasome. We demonstrate that the ability of EEL-1/HUWE1 to safeguard protein turnover requires the HECT-type ubiquitin ligase activity, supporting a model in which EEL-1 ensures degradation via substrate ubiquitination. EEL-1 contains extensive lysine-deficient regions, found at conserved locations in its substrate-binding arena. Through unbiased mutagenesis screening and precise engineering of the EEL-1 protein, we uncover that introducing lysine residues into these regions is detrimental to UPS function and to animal physiology. Together, our findings indicate a central and evolutionarily ancient role for EEL-1/HUWE1 in maintaining optimal UPS function and support targeting this E3 for therapeutic manipulation.

## Author summary

Cells must constantly destroy proteins that are damaged, abnormal, or no longer needed. This process is essential for human health; failure to remove damaged or abnormal proteins contributes to many diseases including age-dependent neurodegenerative disorders. We aimed to understand how cells ensure this system works efficiently, even when it is placed under stress. Using a genetic strategy in the microscopic nematode worm *C. elegans*, we found that a highly conserved protein called EEL-1 (which is equivalent to a protein called HUWE1 in humans) plays a role to help ensure efficient protein destruction when the system is challenged. Large parts of the EEL-1 protein – in regions called lysine deserts - lack the amino acid lysine. We found that, like other proteins involved in protein removal, lysine deserts are important for EEL-1 function. In fact, introducing lysine into its lysine desert causes self-destruction of EEL-1 through the very protein destruction system that it helps operate. Our results shed new light on how EEL-1 helps cells maintain efficient protein destruction, which may be useful for future efforts to develop treatments for diseases in which this process fails.

## Introduction

Rapid and selective protein turnover is needed for cells to maintain protein quality control via removal of damaged and misfolded proteins, and to remodel the proteome in response to environmental changes or developmental cues. Most selective protein degradation is carried out by the ubiquitin proteasome system (UPS). In eukaryotes, the canonical features of the UPS are highly conserved. Proteins are marked for degradation by post-translational conjugation of the small protein ubiquitin (Ub) to lysine residues via isopeptide linkages, a process facilitated by ubiquitin ligases [1]. Ub itself undergoes ubiquitination leading to the synthesis of substrate-anchored polyubiquitin chains [2]. Polyubiquitinated proteins are recruited to the proteasome, an elaborately regulated 33-subunit protease, which is responsible for their proteolytic destruction [3]. The UPS is precisely regulated to ensure cellular protein homeostasis is maintained across differing cell types and to allow dynamic adaptation during developmental transitions or following environmental challenges. However, the regulatory logic by which this is achieved is not fully understood [4]. Misregulation of the UPS is a common feature of disease and a hallmark of aging [5,6]. As such, improved understanding of the UPS regulatory network may lead to new strategies for disease treatment or prevention [7–9].

The SKN-1A/Nrf1 transcription factor is the master regulator of proteasome biogenesis in animal cells and an essential component of the UPS regulatory network [10]. SKN-1A/Nrf1 is required for adequate proteasome subunit gene expression and can boost proteasome subunit biogenesis, allowing cells to compensate for impaired proteasome function or an increased burden of protein misfolding [11–14]. Deglycosylation of SKN-1A/Nrf1 by PNG-1/NGLY1 (a cytosolic peptide:N-glycanase enzyme) is essential for the function of the SKN-1A/Nrf1 pathway [13–15]. In *C. elegans* animals lacking either SKN-1A or PNG-1, proteasome levels are reduced, causing sensitivity to proteotoxic stress, age-dependent defects in tissue homeostasis, and reduced lifespan [14–19]. In humans, the rare genetic disease NGLY1 deficiency causes inactivation of Nrf1, and a range of symptoms associated with proteasome dysfunction [20–23].

EEL-1/HUWE1/Tom1 is an evolutionarily conserved HECT-domain E3 ubiquitin ligase, involved in the degradation of many proteins linked to a wide array of cellular, developmental, and physiological processes [24–45]. The considerable functional versatility of this ligase may be achieved through modular substrate selection; the C-terminal HECT-domain is adjacent to a giant ring-shaped arena that is decorated by several substrate-binding modules, allowing HUWE1 to select diverse substrate proteins for ubiquitination [46–48]. HUWE1 is often mutated and/or overexpressed in cancers and many HUWE1 targets are functionally implicated in tumorigenesis [49]. Further, mutations in HUWE1 cause a severe neurodevelopmental syndrome [50–52] and HUWE1 has been implicated in clearance of protein aggregates and aggregation-prone proteins associated with neurodegenerative diseases [25,26,45]. These connections to various diseases make HUWE1 a promising candidate for therapeutic manipulation, but a deeper understanding of how HUWE1 functions are orchestrated *in vivo* is still needed.

In a mutagenesis screen for genetic interactors of the Nrf1 pathway, we identified numerous alleles affecting *eel-1*, the *C. elegans* HUWE1/Tom1 ortholog. This finding suggests that EEL-1/HUWE1-mediated ubiquitination and SKN-1A/Nrf1-dependent transcriptional control of proteasome levels represent independent yet complementary mechanisms that promote optimal UPS function. Consistently, we found that HUWE1/EEL-1 promotes degradation of a model proteasome substrate, particularly when proteasomal proteolysis is impaired. This activity requires the HECT ubiquitin ligase domain of EEL-1, supporting a role for EEL-1-mediated ubiquitination in maintaining efficient protein turnover. Interestingly, EEL-1/HUWE1, like many proteins connected to UPS function, contains enigmatic ‘lysine deserts’- extended regions that are entirely devoid of lysine residues. Here, we demonstrate that this absence of lysine is critical for EEL-1 function. Specifically, autoubiquitination of EEL-1/HUWE1 regulates its abundance, and when lysines are introduced into these lysine desert regions, the resulting excess autoubiquitination causes a dramatic loss of EEL-1/HUWE1 stability and function. Collectively, our findings identify EEL-1/HUWE1 as a key component within the UPS regulatory network that enables optimal protein turnover. This work suggests that manipulation of HUWE1 activity or specificity could be widely applied to ameliorate UPS dysfunction in disease.

## Results

### EEL-1/HUWE1 promotes degradation of ubiquitin-fused GFP

Reporter proteins fused at their N-terminus to the G76V mutant form of Ub are subject to rapid degradation via the Ub fusion degradation (UFD) pathway and provide a powerful tool to study proteasome-mediated degradation *in vivo* [53–57]. UFD depends on multiple Ub ligases that act in concert to extend Ub chains on the fused ubiquitin moiety [56,58–63]. UFD is remarkably robust to inhibition of the proteolytic capacity of the proteasome; there is no detectable accumulation of Ub[G76V]-fused GFP in cancer cells unless >80% of proteasome activity is eliminated [53]. These observations suggest that the UPS is highly robust to proteolytic challenges. However, the mechanistic basis and physiologic significance of robust UPS function in this context is not clear.

**Fig 1:**
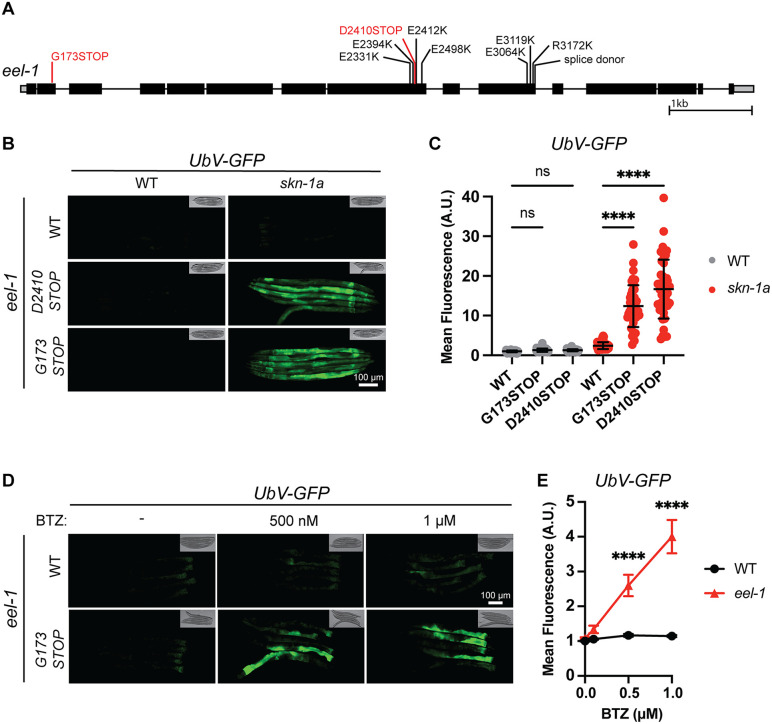
EEL-1/HUWE1 promotes turnover of UbV-GFP. **A)** Schematic of the *eel-1* gene. Locations and effects of mutations identified in a mutagenesis screen for increased accumulation of UbV-GFP are labelled in black. Locations and effects of CRISPR/Cas9-engineered alleles are labelled in red. **B)** Fluorescence micrographs showing that simultaneous inactivation of *skn-1a* and *eel-1* causes dramatic accumulation of UbV-GFP. All images are of L4 stage animals. Scale bar 100 μM. **C)** Quantification of UbV-GFP levels shown in **(B)**. UbV-GFP accumulation is not increased in *eel-1* single mutants and is moderately increased in *skn-1a* single mutants. UbV-GFP accumulation is dramatically increased in *eel-1 skn-1a* double mutants. Error bars show mean ± SD. n = 45 animals per genotype. Ns p > 0.05, **** p < 0.0001, ordinary one-way ANOVA with Šídák’s multiple comparisons test. **D)** Fluorescence micrographs showing that accumulation of UbV-GFP following proteasome inhibition is increased in *eel-1* mutants. L4 animals were exposed to the indicated BTZ concentration (or DMSO control) for 24 hours prior to imaging. Imaged animals are day 1 adults. Scale bar 100 μM. **E)** Quantification of UbV-GFP levels following BTZ exposure as described in panel **(D)**. Exposure of *eel-1* mutant animals to 0.5-1 μM BTZ leads to increased accumulation of UbV-GFP but has no effect on the wild type. Error bars show mean ± SD. N = 30 animals per genotype and condition (BTZ concentrations tested were: DMSO control, 0.1 μM, 0.5 μM, 1 μM). P-values indicate comparison between *eel-1* mutant and wild type for each drug concentration. Ns p > 0.05, **** p < 0.0001, ordinary two-way ANOVA with Šídák’s multiple comparisons test.

We previously generated a transgene that ubiquitously expresses Ub[G76V]::GFP (hereafter UbV-GFP) in *C. elegans* [14,16]. UbV-GFP fluorescence is not detectable in wild type animals but accumulates to low but detectible levels in intestinal cells of *skn-1a* and *png-1* mutant animals, reflecting a requirement for SKN-1A/Nrf1 for adequate proteasome levels [14]. We hypothesized that UbV-GFP accumulation in SKN-1A pathway mutants would be exacerbated if other components that promote robust UPS function are inactivated. To uncover these factors, we carried out a forward genetic screen for increased UbV-GFP fluorescence in animals lacking either *skn-1a* or *png-1*. This screen identified several alleles affecting the *eel-1* gene (Fig 1A). *eel-1* encodes a highly conserved ~465 kDa HECT-domain E3 ubiquitin ligase orthologous to human HUWE1 and *S. cerevisiae* Tom1. HUWE1 knockdown in human cancer cells causes increased accumulation of UbV-GFP [58] and inactivation of Tom1 in yeast causes partial stabilization of UFD substrates [60]. RNAi-mediated knockdown of *eel-1* leads to increased UbV-GFP accumulation in *C. elegans* using an independently generated UbV-GFP transgene [55]. Thus, identification of *eel-1* in our screen supports that this ubiquitin ligase plays a conserved role in degradation of Ub fusion proteins.

To validate our screening results, we used CRISPR/Cas9 to introduce a premature termination codon/frameshift cassette at two positions in the *eel-1* coding sequence (G173STOP, D2410STOP; Fig 1A). Both alleles are expected to act as nulls, given that they were engineered to include a frameshift downstream of the early stop codon and nonsense mediated decay is likely to prevent expression of the truncated protein. In addition, both alleles lack all regions encoding known domains important for EEL-1 function, including the HECT domain. Strikingly, in a wild type genetic background, neither allele causes detectable accumulation of UbV-GFP fluorescence. However, in a *skn-1a* or *png-1* mutant background, *eel-1* inactivation causes very high levels of UbV-GFP, far exceeding levels observed in single mutants (Figs 1B-1C; S1). This suggests that EEL-1 is not essential for proteasomal degradation of UbV-GFP but becomes salient when SKN-1A-mediated control of proteasome levels is lost. Since both premature termination codon mutations have the same effect on UbV-GFP, we used the G173STOP allele for all subsequent experiments.

The genetic interaction between *eel-1* and *skn-1a* suggested that EEL-1 could play an important role to ensure turnover of ubiquitin fusion proteins when proteasome levels or function is reduced. The proteasome inhibitor bortezomib (BTZ) impairs proteasome function by blocking the β5/chymotrypsin-like active site, which is rate-limiting for protein turnover [64,65]. In WT animals, exposure to BTZ at high concentrations causes accumulation of UbV-GFP [16]. We found that in animals lacking EEL-1, the concentration of BTZ required to cause accumulation of UbV-GFP, as measured by live imaging, is significantly lower compared to the wild type (Fig 1D-1E). We conclude that EEL-1 is important to ensure turnover of UbV-GFP in contexts where the capacity for proteasome-mediated protein turnover is impaired.

As already mentioned, UFD depends on multiple ubiquitin ligases, including EEL-1/HUWE1/Tom1, which act in concert to poly-ubiquitinate the fused ubiquitin [56,58–63]. By western blot, we did not detect any reduction in ubiquitination of the UbV-GFP species that accumulate in *eel-1* mutants following BTZ challenge or in the *skn-1a* mutant background (S2 Fig). In addition, we found that in the absence of any proteasomal insult, eel-1 mutants accumulate a small amount of UbV-GFP (S2 Fig), a difference undetectable by live imaging (see Fig 1B,1C). Together, these results indicate that the EEL-1 ubiquitin ligase, although not essential for ubiquitination of UbV-GFP, promotes its proteasomal degradation. The striking accumulation of ubiquitinated forms of UbV-GFP in *eel-1 skn-1a* double mutants indicates that in the context of proteasome insufficiency, EEL-1 is needed to ensure turnover even though UbV-GFP is still subject to ubiquitination by other ligases.

### Simultaneous inactivation of EEL-1 and SKN-1A causes severe defects in development and physiology

**Fig 2:**
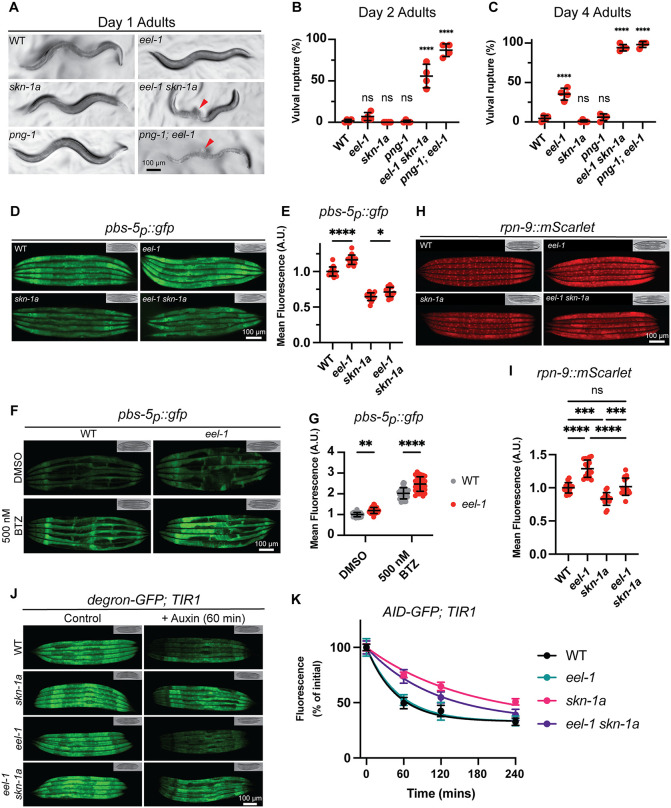
Simultaneous inactivation of EEL-1/HUWE1 and the SKN-1A/Nrf1 pathway causes severe defects in development and physiology that are not caused by reduced proteasome levels. **A)** Micrographs showing day 1 adult animals. Red arrows indicate vulval rupture. Vulval rupture occurs frequently in *eel-1 skn-1a* and *png-1*; *eel-1* double mutants but is not observed in wild type animals or in single mutants. Scale bar 100 μM. **B)** Quantification of vulval rupture in day 2 adults. Results are shown for n = 4 replicate experiments, > 25 animals were assayed in each replicate experiment. Error bars show mean ± SD. Ns p > 0.05, **** p < 0.0001, indicates comparison to the WT control, ordinary one-way ANOVA with Šídák’s multiple comparisons test. **C)** Quantification of vulval rupture in day 4 adults. Results are shown for n = 4 replicate experiments, > 25 animals were assayed in each replicate experiment. Error bars show mean ± SD. Ns p > 0.05, **** p < 0.0001, indicates comparison to the WT control, ordinary one-way ANOVA with Šídák’s multiple comparisons test. **D)** Fluorescence micrographs showing expression of the *pbs-5*_*p*_*::gfp* transcriptional reporter of proteasome subunit gene expression. Expression of the reporter is increased in *eel-1* mutant animals and reduced in *skn-1a* mutant animals. Expression of the reported is not further reduced in *eel-1 skn-1a* double mutants. Images show L4 stage animals. Scale bar 100 μM. **E)** Quantification of *pbs-5*_*p*_*::gfp* reporter expression shown in **(D)**. Expression of the reporter is increased in *eel-1* mutant animals compared to the wild type. Expression is reduced in *skn-1a* mutants. Although expression in *eel-1 skn-1a* double mutants is reduced compared to wild type, it is slightly increased compared to *skn-1a* single mutants. Results are shown for n = 15 animals. Error bars show mean ± SD. * p < 0.05, **** p < 0.0001, ordinary one-way ANOVA with Tukey’s multiple comparisons test. **F)** Fluorescence micrographs showing expression of the *pbs-5*_*p*_*::gfp* transcriptional reporter of proteasome subunit gene expression following exposure to BTZ. L4 animals were exposed to 500 nM BTZ (or DMSO control) for 24 hours prior to imaging. Imaged animals are young adults. Expression of the *pbs-5*_*p*_*::gfp* reporter is increased in *eel-1* mutants as compared to the wild type under both control (DMSO) conditions and following BTZ exposure. Scale bar 100 μM. **G)** Quantification of *pbs-5*_*p*_*::gfp* reporter expression shown in **(F)**. Expression of the *pbs-5*_*p*_*::gfp* GFP reporter is higher in *eel-1* mutants in control conditions and following BTZ exposure. Results are shown for n = 30 animals for each genotype/condition. Error bars show mean ± SD. ** p < 0.01, **** p < 0.0001, ordinary two-way ANOVA, uncorrected Fisher’s LSD. **H)** Fluorescence micrographs showing levels of an endogenously tagged proteasome subunit, RPN-9, tagged with mScarlet. Proteasome levels are increased in *eel-1* mutants and reduced in *skn-1a* mutants but are not further reduced in *eel-1 skn-1a* double mutants. Images show L4 stage animals. Scale bar 100 μM. **I)** Quantification of levels of the RPN-9 proteasome subunit shown in **(H)**. RPN-9 levels are increased in *eel-1* mutants and reduced in *skn-1a* mutants. RPN-9 levels are unchanged compared to the wild type in *eel-1 skn-1a* double mutants. Results are shown for n = 15 animals per genotype. Error bars show mean ± SD. Ns p > 0.05, *** p < 0.001, **** p < 0.0001, ordinary one-way ANOVA with Šídák’s multiple comparisons test. **J)** Fluorescence micrographs showing auxin-induced degradation of degron-GFP. Images show L4 stage animals either before, or 60 minutes after, transfer to plates supplemented with 50 mM auxin. Auxin-induced degradation of degron-GFP is reduced in *skn-1a* mutants but not in *eel-1* mutants. **K)** Quantification of degron-GFP fluorescence following auxin exposure under the same conditions shown in **(J)**; animals were imaged over a 4-hour period following auxin exposure. Destruction of degron-GFP is delayed in *skn-1a* mutants but is not affected by *eel-1*. For each genotype, fluorescence intensity is normalized to the initial (pre-auxin-exposure) value to calculate the percent of fluorescence remaining at each time point. N = 40-45 animals per genotype at each time-point. Error bars show mean ± SD. Trend line shows non-linear regression (one phase decay). degron-GFP half-life in each genotype (95% confidence interval, mins): WT: 28-38; *skn-1a*: 101-121; *eel-1*: 28-44; *eel-1 skn-1a*: 66-88. In all panels of this figure, *eel-*1 denotes the *eel-1*[*G173STOP*] mutant.

Inactivation of EEL-1 causes a range of developmental and physiological defects [39–42]. During our analysis, we noticed that adverse consequences of *eel-1* inactivation are severely exacerbated by loss of the SKN-1A/Nrf1 pathway. In *eel-1 skn-1a* or *png-1*; *eel-1* double mutants, we observed frequent vulval rupture during the first two days of adulthood (Fig 2A, 2B). Additional rupture events occur later and almost all double mutant animals rupture by day 4 (Fig 2C). In contrast, single mutants lacking *eel-1* rarely rupture within the first two days of adulthood, but do show rupture later in adulthood, affecting ~30% of animals by day 4 (Fig 2A-2C). *skn-1a* and *png-1* mutants show elevated vulval rupture later in life (by day 7) [16], but no rupture during the first 4 days of adulthood (Fig 2A-2C). Thus, EEL-1 and SKN-1A each contribute to maintenance of vulval integrity during adulthood and simultaneous inactivation of both is synergistically detrimental. In most *png-1; eel-1* mutant animals, the vulva ruptures prior to significant progeny production and the progeny produced rarely develop into viable adults. As a result, we could not maintain a double mutant population for more than one generation and have not further characterized genetic interactions between *png-1* and *eel-1*.

We also noticed that *eel-1* mutants show a marked delay in larval growth and development, a defect that is particularly severe at elevated temperatures (S3 Fig). *skn-1a* single mutants develop at the same rate as the wild type, but the growth delay caused by *eel-1* inactivation is strongly enhanced in double mutants (S3 Fig). These data indicate that *eel-1* is required for timely developmental progression, and *skn-1a* becomes necessary to support development when *eel-1* is inactive. We conclude that abnormalities in development and physiology caused by inactivation of the EEL-1 ubiquitin ligase are exacerbated by loss of proteasome regulation by SKN-1A. Given the synergistic impact of *eel-1* and *skn-1a* mutations on UbV-GFP levels, these data suggest that simultaneous loss of EEL-1/HUWE1 and SKN-1A/Nrf1 severely impairs the UPS, causing major developmental and physiological defects.

### EEL-1 is not required to maintain adequate proteasome levels

The strong genetic interaction between *eel-1* and *skn-1a* suggests that SKN-1A and EEL-1 could act in separate pathways that independently enhance proteasome levels or activity. To examine whether EEL-1 impacts transcriptional control of proteasome levels, we generated a reporter in which the promoter of the *pbs-5* gene drives GFP expression (*pbs-5*_*p*_*::gfp)*. The *pbs-5* gene encodes the β5 subunit of the proteasome. PBS-5/β5 is essential for proteasome assembly and function, is regulated by SKN-1A, and is likely rate-limiting for proteasome function *in vivo* [14,17,66–69]. We find that loss of *eel-1* does not reduce, but rather increases, *pbs-5*_*p*_*::gfp* expression and does not further decrease *pbs-5*_*p*_*::gfp* expression in *skn-1a* mutants (Fig 2D-2E). Exposure to BTZ leads to increased *pbs-5*_*p*_*::gfp* expression in wild type animals, as expected, and the extent of activation is higher in *eel-1* mutants (Fig 2F-2G). Therefore, the dramatic UbV-GFP accumulation in *eel-1 skn-1a* double mutants is not explained by reduced expression of proteasome subunit genes.

EEL-1 could impact proteasome levels via a post-transcriptional effect not captured by the *pbs-5*_*p*_*::gfp* reporter. We therefore examined animals in which an endogenous proteasome subunit is tagged with mScarlet (*rpn-9::mScarlet*) [70]. Supporting observations made with the *pbs-5*_*p*_*::gfp* transcriptional reporter for proteasome subunit gene expression, mScarlet-tagged RPN-9 protein levels are increased in *eel-1* mutant animals compared to the wild type (Fig 2H, 2I). As expected, *skn-1a* mutants show reduced RPN-9 levels [14], but levels are not further reduced in the double mutants lacking both EEL-1 and SKN-1A (Fig 2H, 2I). Thus, the strong genetic interaction between *eel-1* and *skn-1a* is not explained by a change in proteasome abundance.

### EEL-1 is not generally required for protein degradation by the proteasome

We next sought to address the possibility that proteasome function is generally impaired in animals lacking EEL-1. In western blots for total ubiquitin conjugates, we observed an increase in accumulation of ubiquitinated proteins in *skn-1a* mutant animals, as expected given their reduced proteasome levels. In contrast, we did not observe any increase caused by *eel-1* inactivation, either alone or in combination with *skn-1a* (S4 Fig). Thus, these data suggest that EEL-1 loss does not cause global impairment in the clearance of ubiquitinated proteins by the proteasome.

As an additional test, we examined auxin-induced degradation (AID) of GFP. AID is a heterologous protein degradation system derived from plant hormone signaling and has been used to drive conditional proteasome-dependent protein depletion [71,72]. GFP fused to an auxin-sensitive degron (degron-GFP; expressed in all tissues) is degraded by the proteasome following addition of auxin, triggering interaction with the ubiquitin ligase TIR1. In this system, exposure to 50 mM auxin leads to rapid destruction of degron-GFP, with diminished fluorescence readily detectable after 1 hour [73]. Consistent with reduced proteasome levels causing a general impairment of protein turnover, clearance of degron-GFP is delayed in *skn-1a* mutant animals, with a roughly 3-fold increase in degron-GFP half-life (Fig 2J, 2K). In contrast, auxin-induced degradation is unchanged in *eel-1* mutants. Additionally, there is no further delay in degron-GFP turnover in *eel-1 skn-1a* double mutants compared to *skn-1a* (Fig 2J, 2K). We conclude that EEL-1 is not required for AID of GFP. Since AID is proteasome-dependent, this argues that the strong genetic interaction between *eel-1* and *skn-1a* is not explained by a general reduction in all proteasome functions.

### EEL-1 ubiquitin ligase activity promotes turnover of UbV-GFP and vulval integrity

**Fig 3:**
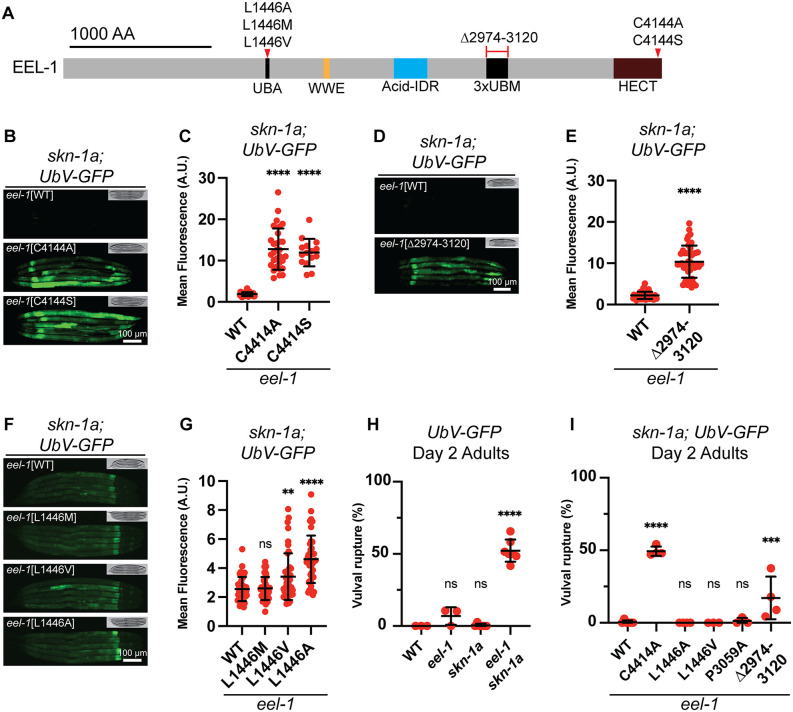
EEL-1/HUWE1 ubiquitin ligase activity is required to promote turnover of UbV-GFP and vulval integrity. **A)** Schematic of the EEL-1 protein showing domain architecture and locations of mutations studied in this figure. **B)** Fluorescence micrographs showing that mutation of the EEL-1 active site causes accumulation of UbV-GFP in a *skn-1a* mutant background. Images show L4 stage animals. Scale bar 100 μM. **C)** Quantification of UbV-GFP levels shown in **(B)**. All animals are *skn-1a* mutant; the x-axis label indicates the *eel-1* genotype. Results are shown for n ≥ 15 animals per genotype. Error bars show mean ± SD. **** p < 0.0001, indicates P-value for comparison to the *eel-1*[WT] control, ordinary one-way ANOVA with Dunnett’s multiple comparisons test. **D)** Fluorescence micrographs showing that deletion of the EEL-1 3xUBM module causes accumulation of UbV-GFP in a *skn-1a* mutant background. Scale bar 100 μM. **E)** Quantification of UbV-GFP levels shown in **(D)**. All animals are *skn-1a* mutant; the x-axis label indicates the *eel-1* genotype. Results are shown for n = 45 animals per genotype. Bars show mean ± SD. **** p < 0.0001, indicates P-value for comparison to the *eel-1*[WT] control, Welch’s two-tailed t test. **F)** Fluorescence micrographs showing the effects of mutation of the EEL-1 UBA domain on UbV-GFP accumulation in a *skn-1a* mutant background. The L1446M mutation is a conservative substitution that humanizes EEL-1 at this residue. The L1446V mutation is equivalent to a patient allele, and the L1446A mutation is expected to disrupt ubiquitin binding similarly to L1446V. UbV-GFP levels are slightly increased in L1446V and L1446A mutant animals. Scale bar 100 μM. **G)** Quantification of UbV-GFP levels shown in **(F)**. All animals are *skn-1a* mutant; the x-axis label indicates the *eel-1* genotype. Results are shown for n = 45 animals per genotype. Error bars show mean ± SD. Ns p > 0.5, ** p < 0.01, **** p < 0.0001, indicates P-value for comparison to the *eel-1*[WT] control, ordinary one-way ANOVA with Dunnett’s multiple comparisons test. **H)** Quantification of vulval rupture phenotypes caused by *skn-1a* and *eel-1* null mutations in animals carrying the UbV-GFP transgene. The *eel-1* mutation tested is *eel-1*[*G173STOP*]. Inactivation *eel-1* and *skn-1a* has the same effect on vulval integrity in the UbV-GFP transgenic background as in non-transgenic animals (compare to Fig 2B). Results are shown for n = 3-7 replicate experiments; 25-35 animals were assayed in each replicate experiment. Error bars show mean ± SD. Ns p > 0.05, **** p < 0.0001, indicates P-value for comparison to the *eel-1*[WT] control, ordinary one-way ANOVA with Šídák’s multiple comparisons test. **I)** Quantification of vulval rupture phenotypes caused by various *eel-1* mutations. All animals are *skn-1a* mutant and carry the UbV-GFP transgene, the x-axis label indicates the *eel-1* genotype. Results are shown for n = 3-4 replicate experiments; 25-35 animals were assayed in each replicate experiment. Error bars show mean ± SD. Ns p > 0.05, *** p < 0.001, **** p < 0.0001, indicates P-value for comparison to the *eel-1*[WT] control, ordinary one-way ANOVA with Šídák’s multiple comparisons test. Additional statistical analysis of these data is provided in S1 Table.

We sought to understand the mechanism by which EEL-1 promotes degradation of UbV-GFP. EEL-1/HUWE1 contains a conserved C-terminal HECT domain responsible for transfer of ubiquitin to substrates. The ubiquitination reaction requires a conserved cysteine residue found within the HECT domain [74]. We engineered inactivating mutations in the EEL-1 HECT domain by CRISPR/Cas9 (C4144A and C4144S) (Fig 3A). These mutations dramatically enhance UbV-GFP accumulation in *skn-1a* mutants (Fig 3B, 3C). We therefore conclude that the E3 ubiquitin ligase activity of EEL-1 is required to promote UbV-GFP turnover, consistent with a conserved role of EEL-1/HUWE1/Tom1 as a ubiquitin ligase in the UFD pathway [58,60].

EEL-1/HUWE1/Tom1 contains conserved ubiquitin-binding domains including a ubiquitin associated (UBA) domain and three repeated ubiquitin binding motifs (UBMs) (Fig 3A). Our screen isolated two alleles (E3064K, E3119K) affecting the UBMs. We noted that the E3064K mutation alters a conserved residue within EEL-1’s second UBM (S5 Fig), near an LP dipeptide required for Ub binding [75,76]. To directly test whether ubiquitin-binding is important for EEL-1 function, we first generated a CRISPR/Cas9 allele, P3059A, which is predicted to disrupt Ub-binding [75]. However, P3059A had no effect on UbV-GFP turnover in *skn-1a* mutant animals (S6 Fig). This suggested that the 3xUBM module may not be required for EEL-1 function in UbV-GFP turnover, or the mutation does not fully disrupt its function. We therefore generated an in-frame deletion allele that removes all three UBMs (Fig 3A). This *eel-1*[*∆2974–3120*] mutation strongly enhances accumulation of UbV-GFP (Fig 3D, 3E), suggesting that robust UbV-GFP turnover requires the 3xUBM Ub-binding domain of EEL-1. Although a similar deletion has been shown to have minimal effects on activity of a fungal ortholog *in vitro* [46], we cannot exclude the possibility that the 3xUBM deletion may alter the overall structure or activity of EEL-1.

A mutation that disrupts the UBA domain of HUWE1, M1328V, causes a neurodevelopmental syndrome [51,77]. The equivalent residue in *C. elegans* EEL-1 is L1446; M1328/L1446 lies within the conserved binding interface through which various UBA domains interact with ubiquitin [78–80]. A conserved mode of Ub-binding involving M1328/L1146 is supported by sequence alignment and Alpha Fold structural prediction (S7 Fig). We generated animals harboring the patient-equivalent mutation L1446V and another substitution L1146A, both of which are expected to disrupt Ub binding. We additionally generated a strain in which the UBA domain of *C. elegans* EEL-1 is ‘humanized’ by a L1446M substitution, which is not expected to disrupt Ub binding. The EEL-1[L1446A] and EEL-1[L1446V] alleles both cause a mild but significant increase in accumulation of UbV-GFP in *skn-1a* mutant animals, whereas there is no effect of the EEL-1[L1446M] substitution (Fig 3F, 3G). Thus, the UBA domain of EEL-1 contributes to robust UbV-GFP turnover, though the effect is modest compared to deletion of the 3xUBM domain. These data suggest that Ub-binding domains of EEL-1 are involved in ensuring optimal degradation of UbV-GFP, consistent with a model in which EEL-1 binds UbV-GFP via Ub-binding domains and promotes UbV-GFP degradation via ubiquitin-dependent ubiquitin ligase activity [58,60].

We next asked whether the *eel-1* alleles affecting the ubiquitin ligase or Ub-binding domains have effects on vulval rupture. We carried out these experiments using animals harboring the UbV-GFP transgene, which we confirmed does not alter the rupture phenotypes caused by *eel-1* and/or *skn-1a* inactivation (Fig 3H; compare to Fig 2B). In a *skn-1a* mutant background, the C4144A HECT domain mutation caused vulval rupture to a similar extent as the *eel-1* null, while mutations affecting ubiquitin binding domains corresponded to their effects on UbV-GFP: only *eel-1*[*∆2974–3120*] showed significant vulval rupture (Fig 3I). Thus, EEL-1 ensures turnover of UbV-GFP and promotes vulval integrity via similar mechanisms, suggesting that ubiquitin ligase activity of EEL-1/HUWE1 plays important roles in animal physiology by promoting optimal protein turnover.

### The Acid-IDR of EEL-1 is not required for degradation of UbV-GFP or vulval integrity

**Fig 4:**
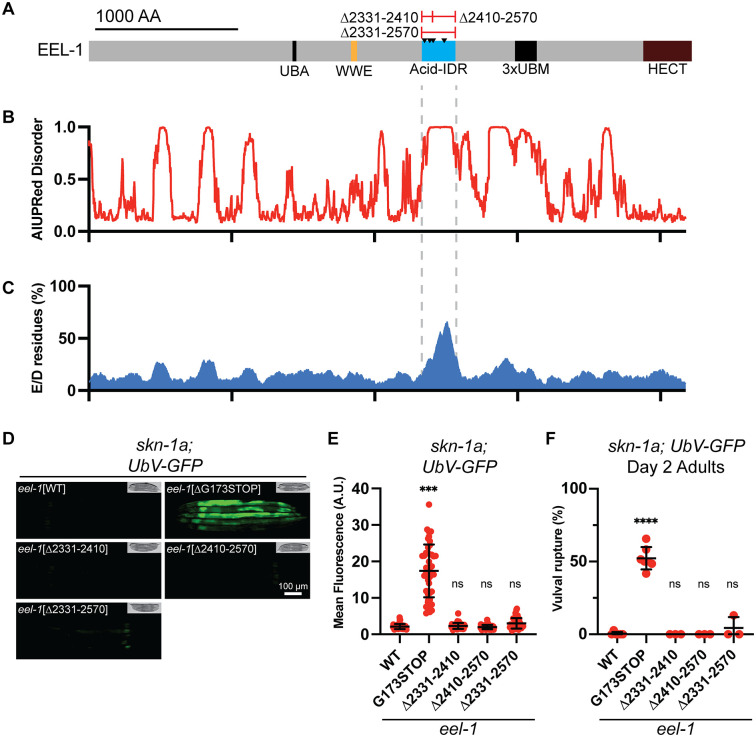
The Acid-IDR domain of EEL-1/HUWE1 is not required for UbV-GFP turnover or vulval integrity. **A)** Schematic of the EEL-1 protein showing domain architecture and locations of in-frame deletion mutations studied in this figure. Black arrows indicate locations of amino acid substitutions identified in our screen for increased UbV-GFP accumulation (E2331K, E2394K, E2412K, E2498K). **B)** The EEL-1 Acid-IDR corresponds to a region of predicted disorder as calculated by AIUPred. **C)** Amino acid composition of EEL-1, showing enrichment of acidic residues in the Acid-IDR. The percent acidic amino acids (glutamate or aspartate) in a 100 amino acid window around each position is plotted. **D)** Fluorescence micrographs showing that deletion of the Acid-IDR of EEL-1 does not lead to increased accumulation of UbV-GFP in *skn-1a* mutants. The G173STOP mutant is included as a positive control. Scale bar 100 μM. **E)** Quantification of UbV-GFP levels shown in **(D)**. All animals are *skn-1a* mutants; the x-axis label indicates the *eel-1* genotype. Results are shown for n = 45 animals per genotype. Error bars show mean ± SD. Ns p > 0.5, *** p < 0.001, indicates P-value for comparison to the *eel-1*[WT] control, ordinary one-way ANOVA with Dunnett’s multiple comparisons test. **F)** Quantification of vulval rupture phenotypes caused by various *eel-1* mutations affecting the Acid-IDR domain. All animals are *skn-1a* mutants and carry the UbV-GFP transgene; the x-axis label indicates the *eel-1* genotype. Results with the *eel-1*[*G173STOP*] allele are included for comparison. Results are shown for n = 3-7 replicate experiments; 25-35 animals were assayed in each replicate experiment. Error bars show mean ± SD. Ns p > 0.05, **** p < 0.0001, indicates P-value for comparison to the *eel-1*[WT] control, ordinary one-way ANOVA with Šídák’s multiple comparisons test. Additional statistical analysis of these data is provided in S1 Table.

Our screen isolated a cluster of amino acid substitutions that do not affect ubiquitin binding domains or the HECT domain (E2331K, E2394K, E2412K, E2498K; Fig 4A). These substitutions cluster within a region of the protein that is highly enriched in acidic residues (~50% glutamate or aspartate) and is intrinsically disordered (Fig 4A-4C). We therefore term this region the Acid-IDR domain. The Acid-IDR domain is involved in recognition of orphaned ribosomal subunits and other basic proteins for ubiquitination and degradation [24,46]. The alleles isolated in our screen each introduce a basic amino acid, lysine, into this domain, which could disrupt electrostatic interactions with substrates or regulators. To test the role of the Acid-IDR, we generated in-frame deletion alleles that remove all or part of the domain (Fig 4A). We observed no UbV-GFP accumulation following deletion of the Acid-IDR in a *skn-1a* mutant background (Fig 4D, 4E). Further, we measured vulval rupture and found no increase compared to control animals (Fig 4F). We conclude that the Acid-IDR of EEL-1 is not required for degradation of UbV-GFP or vulval integrity in *skn-1a* mutant animals.

### Lysine desert mutations in *eel-1* disrupt turnover of UbV-GFP

**Fig 5:**
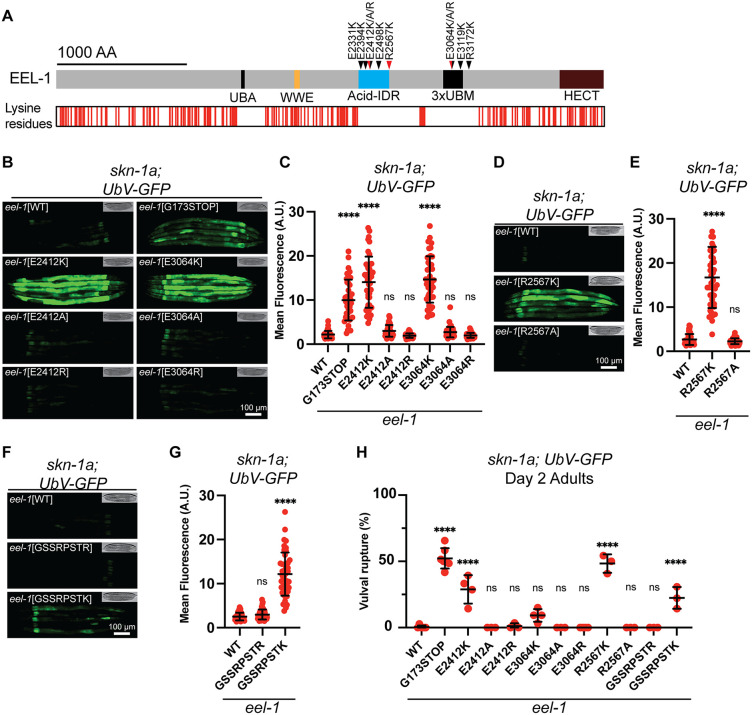
Lysine deficiency within EEL-1/HUWE1 lysine deserts is required for degradation of UbV-GFP and its disruption has varied effects on vulval integrity. **A)** Schematic showing the EEL-1 protein, showing locations of all lysine residues. The Acid-IDR and 3xUBM domains are located within extensive lysine-free regions. Locations of amino acid substitution mutations affecting these domains are indicated. Black arrows indicate mutations isolated via mutagenesis screening; red arrows indicate mutations generated by CRISPR/Cas9 gene editing. **B)** Fluorescence micrographs showing that introducing a lysine (K) at position E2412 or E3064 causes increased accumulation of UbV-GFP in the *skn-1a* mutant background, but that introducing alanine (A) or arginine (R) does not. Scale bar 100 μM. **C)** Quantification of UbV-GFP levels shown in **(B)**. All animals are *skn-1a* mutant; the x-axis label indicates the *eel-1* genotype. Results are shown for n = 45 animals. Error bars show mean ± SD. Ns p > 0.5, ** p < 0.01, **** p < 0.0001, indicates P-value for comparison to the *eel-1*[WT] control, ordinary one-way ANOVA with Dunnett’s multiple comparisons test. **D)** Fluorescence micrographs showing that introducing a lysine (K) at position R2567 causes increased accumulation of UbV-GFP in the *skn-1a* mutant background, but that introducing alanine (A) does not. Scale bar 100 μM. **E)** Quantification of UbV-GFP levels shown in **(D)**. All animals are *skn-1a* mutant; the x-axis label indicates the *eel-1* genotype. Results are shown for n = 45 animals per genotype. Error bars show mean ± SD. Ns p > 0.5, **** p < 0.0001, indicates P-value for comparison to the *eel-1*[WT] control, ordinary one-way ANOVA with Dunnett’s multiple comparisons test. **F)** Fluorescence micrographs showing that insertion of an arbitrary lysine-free sequence, GSSRPSTR, at position 2410 does not cause increased accumulation of UbV-GFP in the *skn-1a* mutant background, but that introducing a similar lysine-containing sequence, GSSRPSTK, does. Scale bar 100 μM. **G)** Quantification of UbV-GFP levels shown in **(F)**. All animals are *skn-1a* mutant; the x-axis label indicates the *eel-1* genotype. Results are shown for n = 45 animals per genotype. Error bars show mean ± SD. Ns p > 0.5, **** p < 0.0001, indicates P-value for comparison to the *eel-1*[WT] control, ordinary one-way ANOVA with Dunnett’s multiple comparisons test. **H)** Quantification of vulval rupture phenotypes caused by various *eel-1* mutations affecting lysine deserts. All animals are *skn-1a* mutants and carry the UbV-GFP transgene; the x-axis label indicates the *eel-1* genotype. Results with the *eel-1*[*G173STOP*] allele are included for comparison. Results are shown for n = 3-7 replicate experiments; 25-35 animals were assayed in each replicate experiment. Error bars show mean ± SD. Ns p > 0.05, **** p < 0.0001, indicates P-value for comparison to the *eel-1*[WT] control, ordinary one-way ANOVA with Šídák’s multiple comparisons test. Additional statistical analysis of these data is provided in S1 Table.

Interestingly, our unbiased mutagenesis screen identified several amino acid substitutions that introduce lysine (K) residues in two distinct clusters (Fig 5A). The clusters are in or nearby to the Acid-IDR (E2331K, E2394K, E2412K, E2598K) and 3xUBM domains (E3064K, E3119K, R3172K). Comparing the sequence composition and domain organization of EEL-1/HUWE1/Tom1 orthologs across diverse eukaryotes, these domains are positioned within deeply conserved lysine-deficient regions (termed lysine deserts; Figs 5A, S8, S9). Lysine deserts are frequently found in UPS components and prevent their inactivation via accidental ubiquitination [81–86]. We therefore hypothesized that the absence of lysine from these domains is required for EEL-1 function.

We used CRISPR/Cas9 to recreate two of the mutations isolated in our screen; E2412K (a substitution within the Acid-IDR) and E3064K (within the 3xUBMs) (Fig 4A). As expected, both cause increased accumulation of UbV-GFP in the *skn-1a* mutant background (Fig 5B, 5C). Importantly, substitution of alanine (A) or arginine (R) at these positions has no effect (Fig 5B, 5C). These data suggest that disruption of EEL-1/HUWE1 function by these mutations is a specific consequence of introducing lysine to EEL-1’s lysine-free regions.

The lysine deficient regions of EEL-1 span hundreds of amino acids. We wondered whether our screen had pinpointed specific locations within the desert that are particularly sensitive to introduction of a lysine. To test this idea, we arbitrarily selected a basic residue within this region, R2567, which is at the periphery of the Acid-IDR domain but near the center of a lysine desert (Fig 5A). Strikingly, in a *skn-1a* mutant background, R2567K, but not R2567A, causes a strong enhancement of UbV-GFP accumulation (Fig 5D, 5E). The strong effect of introducing a lysine at a randomly selected location suggests that this lysine-deficient region of EEL-1/HUWE1 is intolerant of lysine at many or all positions, as implied by its deep evolutionary conservation.

Our data indicate that ectopic lysine residues in the Acid-IDR of EEL-1 are not tolerated, even though this domain can be deleted without disrupting function. This suggests that the ectopic lysine residues do not simply disrupt an Acid-IDR-dependent function. Rather, EEL-1 function in general requires that this domain is lysine-free. To test this, we inserted a short lysine-free arbitrary sequence, GSSRPSTR, into the Acid-IDR after the 2410^th^ residue (*eel-1*[*PSTR*^*2410*^]), and a similar lysine-bearing variant insertion, GSSRPSTK (*eel-1*[*PSTK*^*2410*^]). Strikingly, only the lysine-bearing insertion causes a UbV-GFP accumulation in a *skn-1a* mutant background (Fig 5F, 5G). Thus, lysine residues, even if engineered to alter a novel sequence not normally present in the protein, still potently disrupt function. This confirms that the need for these regions to be lysine-free includes residues that have no direct relevance to the surrounding domain’s function(s). This suggests that conservation of the lysine-deficient domains is driven by strict selection to be lysine-free, which creates an additional sequence constraint on top of those imposed by the domains’ functions.

### Lysine desert mutations in *eel-1* variably disrupt vulval integrity

To gauge the physiological impact of manipulations to the lysine desert of EEL-1, we measured their effect on vulval integrity in day 2 adults (Fig 5H). In the *skn-1a* genetic background, none of the non-lysine substitutions or insertions caused any increase in vulval rupture, consistent with little or no effect on *eel-1* function. In contrast, mutations that introduce an ectopic lysine cause vulval rupture in the *skn-1a* mutant background, albeit to very varying extents. The variant we engineered at a randomly selected position, R2567K, caused the strongest phenotype, equivalent to an *eel-1* null allele, but the amino acid substitutions isolated in our mutagenesis screen had less dramatic effects (Fig 5H, S1 Table). The E2412K variant causes an intermediate rupture phenotype, whereas we did not observe elevated vulval rupture in E3064K mutants. However, *eel-1*[*E3064K*] *skn-1a* double mutants did display markedly elevated vulval rupture by day 4 of adulthood (S10 Fig). When analyzed as single mutants, neither *eel-1*[*E2412K*] or *eel-1*[*E3064K*] caused vulval rupture at day 4 of adulthood, a phenotype we had observed in the *eel-1* null, suggesting that these mutations do not fully inactivate EEL-1 (Figs 2C, S10). Thus, the physiological impacts of lysine desert mutations are variable and dependent on the specific location at which ectopic lysine is introduced.

### Mutant phenotypes caused by an ectopic lysine residue are rescued by an engineered protein-protein interaction

**Fig 6:**
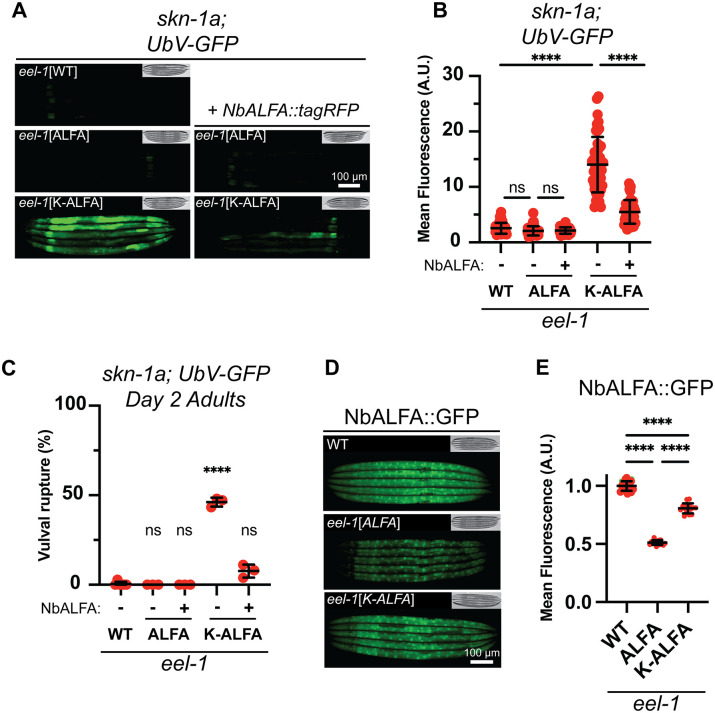
Engineered changes to the lysine desert of EEL-1/HUWE1 modulate UbV-GFP turnover and vulval integrity. **A)** Fluorescence micrographs showing that insertion of a proline-flanked ALFA tag (“ALFA”’; PSRLEEELRRRLTEP) at position 2410 does not cause increased accumulation of UbV-GFP in the *skn-1a* mutant background, but the same insertion accompanied by a flanking lysine residue (“K-ALFA”; KPSRLEEELRRRLTEP), does. The UbV-GFP accumulation caused by the K-ALFA insertion is substantially reduced by expression of NbALFA::tagRFP. Scale bar 100 μM. **B)** Quantification of UbV-GFP levels shown in **(A)**. All animals are *skn-1a* mutant; the x-axis label indicates the *eel-1* genotype and the presence of NbALFA::tagRFP. Results are shown for n = 45 animals per genotype. Error bars show mean ± SD. Ns p > 0.5, **** p < 0.0001, ordinary one-way ANOVA with Tukey’s multiple comparisons test. **C)** Quantification of vulval rupture phenotypes caused by engineered insertion of an ALFA tag in the EEL-1 lysine desert. All animals are *skn-1a* mutants and carry the UbV-GFP transgene; the x-axis label indicates the *eel-1* genotype and presence of NbALFA::tagRFP. Results are shown for n = 3 replicate experiments; 25-35 animals were assayed in each replicate experiment. Error bars show mean ± SD. Ns p > 0.05, **** p < 0.0001, indicates comparison to the eel-1[WT] control, ordinary one-way ANOVA with Šídák’s multiple comparisons test. Additional statistical analysis of these data is provided in S1 Table. **D)** Fluorescence micrographs showing that engineered insertion of an ALFA tag at position 2410 of *eel-1* leads to degradation of NbALFA::GFP, and that the extent of degradation is limited if the engineered insertion includes an adjacent lysine residue. Scale bar 100 μM. **E)** Quantification of NbALFA::GFP levels shown in **(D)**. The x-axis label indicates the *eel-1* genotype. Results are shown for n = 15 animals per genotype. Error bars show mean ± SD. **** p < 0.0001, ordinary one-way ANOVA with Tukey’s multiple comparisons test.

Lysine deserts are often found in intrinsically disordered domains that are likely to be dynamically exposed to solvent and/or cellular interactors, properties that might predispose adventitious ubiquitination [81]. To investigate the relationship between disorder, solvent exposure, and lysine desert mutations’ functional impact, we inserted a proline-flanked ALFA tag (PSRLEEELRRRLTEP, a lysine-free engineered sequence with high helical propensity [87]) after the 2410^th^ residue of EEL-1 (*eel-1*[*ALFA*^*2410*^]). We generated a second edit in which the same insertion is preceded by a lysine residue (KPSRLEEELRRRLTEP; *eel-1*[*K-ALFA*^*2410*^]). We find that insertion of ALFA into the disordered Acid-IDR does not cause UbV-GFP accumulation in the *skn-1a* mutant background, unless accompanied by insertion of a lysine residue (Fig 6A, 6B). Consistently, in the *skn-1a* mutant background, *eel-1*[*K-ALFA*^*2410*^], but not *eel-1*[*ALFA*^*2410*^], causes vulval rupture (Fig 6C). In fact, the extent of vulval rupture caused by the K-ALFA insertion is higher than that caused by the non-helical GSSRPSTK insertion at the same location. Thus, a short helical insertion in the Acid-IDR does not impair function unless accompanied by an ectopic lysine residue. Moreover, the detrimental effect of an ectopic lysine at this location is not blunted, but rather exacerbated, if adjacent to an engineered helical insertion in the disordered Acid-IDR.

NbALFA, a single chain antibody, binds to the ALFA tag with very high affinity [87]. We wondered whether NbALFA binding would have any effect on the function of EEL-1[ALFA^2410^] or EEL-1[K-ALFA^2410^]. We hypothesized that tightly associating the Acid-IDR, which is disordered and lysine deficient, to a non-disordered and lysine-containing protein, could interfere with EEL-1[ALFA^2410^] function. Alternatively, for EEL-1[K-ALFA^2410^], placing a bulky binding partner adjacent to the ectopic lysine could modulate detrimental effects. We engineered animals to express NbALFA fused to tagRFP under the strong *rpl-28* promoter from a single-copy genomic insertion. Interestingly, NbALFA::tagRFP has no apparent effect on EEL-1[ALFA^2410^] function, as measured by accumulation of UbV-GFP or vulval rupture in a *skn-1a* mutant background (Fig 6A-6C). In contrast, the defect in UbV-GFP turnover and the vulval rupture of *eel-1*[*K-ALFA*^*2410*^] animals is strongly suppressed by NbALFA::tagRFP (Fig 6A-6C). This suggests that the detrimental effects of the ectopic lysine residue in EEL-1[K-ALFA^2410^] is dampened by NbALFA binding, likely by restricting the exposure of the ectopic lysine to the cellular milieu.

### Ectopic lysine impairs degradation of an engineered EEL-1 substrate

In EEL-1[ALFA^2410^]-expressing animals, binding of NbALFA fusion proteins to the ALFA tag would recruit them to the substrate recognition arena of EEL-1 and would likely cause their degradation. We examined the levels of an NbALFA::GFP fusion in *eel-1*[ALFA^2410^] animals and observed a significant reduction, suggesting that the engineered interaction between EEL-1 and NbALFA::GFP causes its ubiquitination and destruction. Interestingly, destabilization of NbALFA::GFP by EEL-1[K-ALFA^2410^] is diminished compared to EEL-1[ALFA^2410^], although both contain an intact ALFA sequence (Fig 6D, 6E). Thus, introduction of a lysine residue within the Acid-IDR domain interferes with degradation of an engineered substrate that binds adjacent to the ectopic lysine residue. These data suggest that the ectopic lysine residue reduces the ability of EEL-1 to ubiquitinate bound substrates and/or trigger their subsequent destruction.

### Lysine desert mutations destabilize EEL-1 in a ligase activity-dependent manner

**Fig 7:**
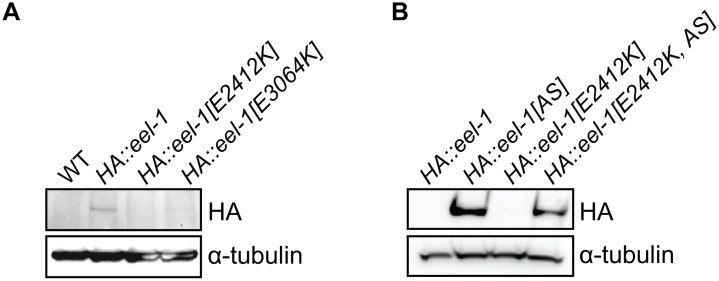
Ectopic lysine residues destabilize EEL-1/HUWE1 via autoubiquitination. **A)** Western blot showing the effect of lysine-introducing mutations on levels of HA-tagged endogenous EEL-1. The E2412K mutation reduces HA::EEL-1 to undetectable levels, and the E3064K mutation reduces HA::EEL-1 to barely detectable levels. **B)** Western blot showing the effect of mutation of the HECT-domain active site (AS; C4144A) on the stability of HA-tagged endogenous EEL-1. The AS mutation causes a massive increase in the levels of wild type HA::EEL-1 and of the E2412K mutant.

Lysine deserts can safeguard against accidental ubiquitination leading to self-destruction of UPS components [81–86]. To address whether lysine amino acid substitutions affect the stability of EEL-1, we engineered E2412K and E3064K substitutions into a strain in which endogenous EEL-1 is tagged with HA (HA::EEL-1; the tag does not disrupt function, S11 Fig). Western blots revealed that HA::EEL-1 levels are significantly reduced in E3064K mutants and undetectable in E2412K mutants, demonstrating that these lysine substitutions destabilize EEL-1 (Fig 7A). Το test whether this destabilization is due to autoubiquitination, we tested the effect of an active site mutation (C4144A) on HA::EEL-1[E2412K] stability. We found that the levels of HA::EEL-1[E2412K, C4144A] increased dramatically compared to HA::EEL-1[E2412K] (Fig 7B), suggesting that lysine-introducing mutations destabilize EEL-1 in a manner dependent on its ubiquitin ligase activity. While performing these experiments we found that the active site mutation causes a massive increase in levels of HA::EEL-1 regardless of the presence of a lysine-introducing mutation (Fig 7B). This suggests that catalytically inactivating EEL-1 increases its stability even in the absence of a lysine-introducing mutation. Taken together, these data suggest that autoubiquitination of EEL-1/HUWE1 normally functions to control its protein levels, but excessive autoubiquitination in lysine desert mutants leads to catastrophic loss of EEL-1/HUWE1 stability and function.

## Discussion

Proteins artificially fused to ubiquitin are subject to proteasomal degradation via the UFD pathway [53,56,57]. Analysis of UFD pathway reporters (such as UbV-GFP) indicate that UFD is remarkably resilient to impairment of proteasome activity. In cancer cells, no accumulation of UbV-GFP is observed in cells even when approximately 80% of proteasomes are inactivated by a proteasome inhibitor drug [53]. Our analysis provides insight into how UFD tolerates proteasomal challenges. Further, our findings provide evidence that this mechanism, together with the SKN-1A/Nrf1 pathway, safeguards UPS function to ensure normal animal development and physiology.

In UFD and in protein quality control more generally, HUWE1/Tom1 appears to collaborate with other ubiquitin ligases to create ubiquitin chain(s) that cause proteasomal degradation [25,58,60,88,89]. Interestingly, *in vitro* ubiquitination of UFD substrates by Tom1 requires a second UFD ubiquitin ligase, Ufd4, which is orthologous to HECD-1/TRIP12 [60]. Further, HUWE1 preferentially modifies substrates already modified by long ubiquitin chains [25] and interactome studies suggest that HUWE1 binds ubiquitin chains of varied linkage topologies [27,88]. Altogether, these observations suggest that EEL-1/HUWE1/Tom1 primarily acts on pre-ubiquitinated substrates as part of a complex system involving multiple ubiquitin ligases that controls turnover of ubiquitin fusions. A full understanding of this system, and its relevance to the developmental and physiological defects caused by *eel-1* mutations, will require more detailed genetic and biochemical analysis of the collaboration between EEL-1 and other UFD-associated Ub ligases.

Our findings, together with studies of human and fungal orthologs, suggest that EEL-1/HUWE1/Tom1 is particularly important for maintaining proteostasis when cellular capacity for adequate protein turnover is challenged [24–26,60]. How does EEL-1 promote robust protein turnover in this context? One possibility is that EEL-1/HUWE1 acts in association with the proteasome. The ubiquitin ligase Hul5/UBE3C/ETC-1 has been shown to elongate ubiquitin chains on substrates already engaged with the proteasome, thus prolonging their association with the proteasome and promoting degradation [90–94]. HUWE1 has also been reported to associate with the proteasome [58,95,96], raising the possibility that it may similarly enhance the degradation of proteasome-associated substrates. Such a mechanism would be expected to become particularly important when proteasome abundance or activity is limiting. Further studies will be needed to test this model and clarify the precise mechanism(s) by which EEL/HUWE-1 exerts its effects under conditions of proteasomal stress.

Our genetic analysis reveals that simultaneous inactivation of *eel-1* and *skn-1a* causes severe defects in both UFD and the overall health of the animal. These data support a model in which SKN-1A and EEL-1 act in distinct pathways that each support optimal function of the UPS, revealing a crucial role for EEL-1/HUWE1 in the regulatory network that adapts the UPS to fulfill cellular needs. UPS dysfunction is a hallmark of aging and a cause of age-associated loss of proteostasis [5,6]. Age-dependent vulval integrity defects are a marker of loss of tissue homeostasis during aging in *C. elegans* [97]. Our observations show that whereas *eel-1* and *skn-1a* single mutants show elevated vulval rupture in an age-dependent manner [16], double mutants show a severe defect in vulval integrity as young adults. Catastrophic enhancement of this age-dependent phenotype in double mutants reinforces the view that EEL-1/HUWE1 and SKN-1A/Nrf1 act in a complementary manner to maintain proteostasis and animal health during aging. It will be important to identify the proteasomal substrate(s) that underlie the synergistic effects of EEL-1 and SKN-1A inactivation and define their roles in development, physiology, and aging.

UPS dysfunction is linked to abnormalities in human neuronal development, function, and adult-onset neurodegenerative diseases. Mutations affecting the Nrf1 pathway, HUWE1, or proteasomal components themselves cause an overlapping spectrum of neurodevelopmental symptoms [50–52,77,98–107]. Both the SKN-1A/Nrf1 pathway and EEL-1/HUWE1 are protective against aggregation of proteins implicated in late-onset proteinopathies such as Alzheimer’s Disease and Huntington’s Disease [14,16,25,106,108–111]. Pharmacological interventions that increase HUWE1 levels or activity may have therapeutic potential in neurological conditions. Alternatively, manipulating HUWE1 substrate specificity could represent a general avenue for therapeutic manipulation of the UPS.

Protein domains entirely deficient in lysine residues are a conserved feature of eukaryotic proteomes and are frequently found in proteins that participate in UPS function [81]. However, lysine deserts’ relevance to animal development and physiology have been unclear, and their specific functional roles are not well understood. Our genetic analysis demonstrates that the absence of lysine residues from the lysine desert of EEL-1 is important for animal development and physiology. Interestingly, a lysine-introducing mutation in *eel-1* (E2475K, within the Acid-IDR lysine desert) alters neuronal architecture, supporting relevance to neuronal development and function [41].

Our findings represent the first analysis of the role of a lysine desert in a HECT-type E3. The fact that HECT E3s covalently bind Ub before transferring it to the substrate may render this class of enzymes particularly dependent on lysine deserts to avoid autoubiquitination. Almost all human HECT-type ubiquitin ligases contain lysine deserts, supporting this possibility [81]. Ubiquitination within lysine deserts has been suggested to cause degradation [81–86] and our analysis of the E2412K and E3064K mutations also revealed a reduction of EEL-1 levels, indicating that introducing lysine in these regions indeed destabilizes EEL-1. Further analysis of the E2412K mutant show that ligase activity is needed for the destabilization, suggesting that it is caused by autoubiquitination. Interestingly, mutation of the HECT-domain active site also stabilizes wild type EEL-1, suggesting a model in which EEL-1 tunes its own abundance via autoubiquitination. Consistently, autoubiquitination has been detected by *in vitro* assays of EEL-1/HUWE1 orthologs from animals and fungi [46–48,112]. Collectively, these data suggest that autoubiquitination of EEL-1/HUWE1 normally functions to tune EEL-1 protein levels, but excessive autoubiquitination in lysine desert mutants leads to catastrophic loss of EEL-1 function.

Many proteins containing lysine deserts are highly conserved throughout eukaryotic evolution [81]. Lysine deserts are also present in bacterial species that regulate protein turnover via pupylation, suggesting they were an early molecular adaptation to protein degradation via lysine-linked protein conjugation [83]. Inspecting the sequences of EEL-1/HUWE1/Tom1 orthologs reveals deep conservation of domain architecture and sequence compositional biases. This conservation extends to these domains’ structural arrangement, as revealed by experimentally determined structures of human, nematode, and fungal orthologs [46–48]. This implies that the common ancestor of EEL-1/HUWE1/Tom1 contained a HECT domain accompanied by lysine-deficient Ub-binding and Acid-IDR domains approximately 1.5 billion years ago. The ancient origin of EEL-1/HUWE1/Tom1 argues that its present-day functional versatility exists because it has repeatedly been coopted into degradation pathways that must function optimally to support the complexities of eukaryotic biology.

## Methods

### *C. elegans* strains and maintenance

C. ele*gans* were maintained on standard nematode growth media (NGM) at 20°C and fed *E. coli* OP50 unless otherwise noted. A list of strains used in this study is provided in S2 Table. Some strains were provided by the CGC, which is funded by NIH Office of Research Infrastructure Programs (P40 OD010440). Ethane-methyl-sulfonate (EMS) mutagenesis was carried out by treating ~10,000 L4 animals with 47 mM EMS for 4 hours at 20°C. After mutagenesis, large populations of synchronized F2 animals were screened and individuals with increased accumulation of UbV-GFP were isolated. This screen was carried out using *skn-1a*(*mg570*); *mgIs77*[*UbV-GFP*] and in *png-1*(*ok1654*); *mgIs77*[*UbV-GFP*] animals.

### Whole genome sequencing and analysis

Genomic DNA was prepared using the Gentra Purgene Tissue kit (Qiagen, #158689). Genomic DNA libraries were prepared using the NEBNext genomic DNA library construction kit (New England Biolabs, #E6040). Libraries were sequenced using an Illumina HiSeq instrument. Deep sequencing reads were analyzed using a custom Galaxy workflow adapted from CloudMap [113]. Candidate causative alleles were identified by the isolation of multiple independent mutations affecting the same gene.

### Growth rate assay

Embryos were isolated by bleaching of gravid adult animals and then hatched overnight in M9 at 20°C while rotating. The resulting population of synchronized L1 larvae were plated to fresh NGM plates seeded with OP50 and incubated at the desired temperature. After 48–72 hours, plates were imaged using a Leica M165FC equipped with a 910 Leica K5 sCMOS camera and using LAS X software. Animal length was measured using the segmented line tool in ImageJ.

### Vulval rupture assay

To measure adult rupture, 30–40 L4 animals were selected at random from mixed stage cultures and transferred to a fresh plate. The animals were monitored for rupture (indicated by intestinal or germline material outside the vulva) after 48 hours (day 2 adults), and again after a further 48 hours (day 4 adults). Throughout the rupture assay, adults were transferred to fresh plates every 2–3 days to separate them from progeny. To image vulval rupture in day 1 adults, L4 animals were selected at random from mixed stage cultures and transferred to a fresh plate. After 24 hours, the animals were imaged using a Leica M165FC equipped with a 910 Leica K5 sCMOS camera and using LAS X software.

### Plasmid constructs and transgenesis

All cloning was carried out using the NEBuilder HiFi DNA Assembly kit (New England Biolabs #E2621). The sequence encoding NbALFA was codon optimized for expression in *C. elegans* using the transgenebuilder tool (https://www.wormbuilder.org/transgenebuilder/) and synthesized to include a single intron (from the *rps-0* gene) and a C-terminal in-frame linker sequence (STSGGSGGTGGSS). Gene synthesis was carried out by Twist Biosciences. Gibson assembly was used to generate plasmids in which NbALFA fusion proteins are expressed under control of the ubiquitous *rpl-28* promoter (605 bp immediately upstream of the *rpl-28* ORF) and are followed by the *tbb-2* 3’UTR (376 bp immediately downstream of the *tbb-2* stop codon). These constructs were inserted to the pNL43 backbone, to allow generation of transgenics by miniMos [15,114]. pNL559 (Rpl-28p::NbALFA::tagRFP::tbb-2) and pNL549 (Rpl-28p::NbALFA::GFP::tbb-2), were used to generate transgenic (miniMos insertion) animals harboring single-copy transgenes driving ubiquitous expression of NbALFA::tagRFP and NbALFA:tagRFP respectively [114].

The *pbs-5*_*p*_*::gfp* reporter was generated by placing the promoter of CEOP1752 (850 bp upstream of the first open reading frame of the operon, *K05C4.2* (*pbs-5* is the second gene in the operon), upstream of a fragment containing the GFP coding sequence and *tbb-2* 3’UTR (376 bp immediately downstream of the *tbb-2* stop codon), and inserted to the pBlueScript backbone, to generate pNL136 (*pbs-5*_*p*_*::GFP*). High-copy-number arrays containing pNL136 were integrated to chromosome II via CRISPR/Cas9 [115,116].

### Genome Modification by CRISPR/Cas9

CRISPR/Cas9 genome editing was carried out through microinjection of guide RNA/Cas9 ribonucleoprotein (RNP) complexes (IDT #1081058, #1072532, and custom crRNA) along with single-stranded oligonucleotides (IDT) as homology-directed repair (HDR) templates [117,118]. Sequences of all guide RNAs and HDR oligos are provided in S3 Table. All CRISPR edits were identified by diagnostic PCR and confirmed by sequencing.

### Microscopy

Brightfield and fluorescence images of whole animals (UbV-GFP, *pbs-5*_*p*_*::gfp*, *rpn-9::mScarlet*, degron-GFP, NbALFA::GFP) were collected on a Leica M165FC equipped with a 910 Leica K5 sCMOS camera and using LAS X software. For fluorescence imaging, worms were immobilized using sodium azide and mounted on 2% agarose pads. All images were processed and analyzed using ImageJ/Fiji software. Images shown within the same figure panel were collected using the same exposure time and were then processed identically. To quantify accumulation of UbV-GFP, the mean pixel intensity in the anterior intestinal cells was measured. To quantify *pbs-5*_*p*_*::GFP* reporter expression, *rpn-9::mScarlet* levels, degron-GFP levels, and NbALFA::GFP levels, the mean pixel intensity was measured using a polygon selection around each animal.

### Bortezomib treatment

NGM plates containing the desired concentration of bortezomib (LC Laboratories, #B1408) were prepared by directly applying bortezomib solution to NGM plates seeded with OP50 and leaving the plates to dry for 1–2 hours. Control plates supplemented with DMSO were prepared in parallel. L4 stage animals to were shifted to bortezomib or control DMSO-supplemented plates and imaged after 24 hours.

### Western blot

For UbV-GFP western blots: approximately 60 L4 stage animals were placed on regular NGM plates, plates supplemented with 1 μM bortezomib, or control DMSO plates, and incubated overnight at 20°C. The following day, 50 day 1 adults were transferred into microcentrifuge tubes containing 30 μL M9. Then, 15 μL of Bolt LDS sample buffer (Invitrogen; #B0008) was added to each tube. Samples were frozen at -80°C for 15 minutes, followed by incubation at 70°C for 10 minutes. Samples were then vortexed at medium speed for 5 minutes. Then, 4.5 μL of Bond- Breaker TCEP solution (Thermo Scientific; #77720) was added, and samples were incubated at room temperature for 5 minutes. 20–30 μL of each sample was loaded onto protein gels. For HA-tagged EEL-1 and Ub western blots: 50 day 1 adults were transferred into microcentrifuge tubes containing 20 μL M9. Then, 15 μL of NuPAGE LDS sample buffer (Invitrogen; #NP0007) was added to each tube. Samples were frozen at -80°C for 15 minutes, followed by incubation at 70°C for 10 minutes. Samples were then vortexed at medium speed for 5 minutes. Then, 3.5 μL of Bond-Breaker TCEP solution (Thermo Scientific; #77720) was added, and samples were incubated at room temperature for 5 minutes. 25 μL of each sample was loaded onto protein gels.

SDS-PAGE was performed using Bolt 4–12% Bis–Tris Plus gels (Invitrogen, #NW04122BOX) and MOPS SDS running buffer (Invitrogen; #B0001; UbV-GFP, Ub) or NuPAGETM 3–8% Tris-Acetate gels (Invitrogen; #EA0375BOX) and Tris-Acetate SDS running buffer (Invitrogen, #LA0041; HA-tagged EEL-1). Western blotting was performed using iBlot 2 nitrocellulose mini stacks (Invitrogen; #IB23002) or iBlot 2NC regular stacks (Invitrogen; IB23001), according to the manufacturer’s instructions. Membranes were blocked using 5% milk or 4% bovine serum albumin (BSA; Gold-Bio; #A-420–50). The following antibodies were used: anti-GFP mouse monoclonal antibody (SIGMA; 11814460001; 1:1,000 dilution), anti-HA tag (F7) mouse monoclonal antibody (Santa Cruz; #sc-7392; 1:1,000 dilution), anti-ubiquitin mouse monoclonal antibody (Santa Cruz #sc-8017), anti-α-tubulin mouse monoclonal antibody (SIGMA, #T6074; 1:10,000 dilution), and goat anti-mouse IgG antibody, HRP conjugate (Millipore, #12–349; 1:2,500 dilution). The SuperSignal West Pico PLUS chemiluminescent substrate (Thermo Scientific, #34577) or SuperSignal West Dura extended duration substrate (Thermo; #34075) were used for detection of the HRP signal.

### Auxin-induced degradation

NGM plates containing 50 mM auxin (α-Napthaleneacetic acid, Phytotech Labs #N610) were prepared by directly adding auxin solution to NGM plates seeded with OP50 and leaving plates to dry for 1–2 hours. Control (non-auxin-exposed) L4 animals were imaged immediately prior to beginning the auxin treatment. L4 stage animals were shifted to auxin-supplemented plates and imaged after 1, 2 and 4 hours.

### Protein sequence analysis

EEL-1/HUWE1/Tom1 orthologs were identified in the PANTHER database subfamily: E3 UBIQUITIN-PROTEIN LIGASE HUWE1 (PTHR11254:SF67) [119]. Protein sequences of EEL-1 and orthologs were retrieved from Uniprot [120]. Multiple sequence alignment was carried out using Clustal Omega with default settings [121]. AIUPred was used to predict intrinsically disordered regions [122]. Sequence composition was analyzed in excel. Structure prediction was carried out using Alpha Fold 3 and structure predictions were visualized using UCSF ChimeraX [123,124].

### Statistical analysis

Statistical tests were carried out using GraphPad Prism. All biological replicates were performed using independent populations of animals. All numerical data used in graphs and statistical tests are provided in S1 Data File.

## Supporting information

S1 Fig Dramatic accumulation of UbV-GFP in *png-1; eel-1* double mutants. A) Fluorescence micrographs showing accumulation of UbV-GFP in *png-1*; *eel-1* double mutants. All images are of L4 stage animals. Scale bar 100 μM. B) Quantification of UbV-GFP levels shown in (A). UbV-GFP is dramatically increased in *png-1*; *eel-1* double mutants. Error bars show mean ± SD. n > 15 animals per genotype. **** p < 0.0001, Welch’s t test.
(TIF)

S2 Fig Inactivation of EEL-1 does not cause loss of UbV-GFP ubiquitination. Western blot showing UbV-GFP levels and ubiquitination. For each sample, lysates were prepared from 50 (day 1 adult) animals. The first 6 lanes compare animals either exposed to DMSO control or 1 μM BTZ prior to collection (*skn-1a* indicates *skn-1a*[*G2STOP*], and *eel-1* indicates *eel-1*[*G172STOP*]). The final 2 lanes show animals maintained under standard culture conditions (*skn-1a* indicates *skn-1a*[*G2STOP*] allele and *eel-1* mutations as indicated). Inactivation of *eel-1* causes accumulation of a low, barely detectable, level of UbV-GFP, which is not detected in wild type animals (compare lane 1 to lane 3). Accumulation of UbV-GFP is increased following BTZ exposure, without any obvious difference in ubiquitination between the genotypes tested. The *eel-1*[*E2412K*] and *eel-1*[*E3064K*] mutations are generated by CRISPR to recapitulate mutations isolated in our EMS screen. Both mutations cause strong accumulation of UbV-GFP when combined with loss of SKN-1A, again without any evidence of loss of ubiquitination.
(TIF)

S3 Fig Growth defects of *eel-1* mutants are enhanced by *skn-1a* inactivation. A) Images showing the effect of *skn-1a* and *eel-1* mutations on developmental progression. Animals were synchronized at the L1 stage by hatching in buffer overnight and then cultured in the presence of OP50 bacteria for 72 hours at 20ºC. Scale bar 300 μM. B) Quantification of animal length from (A). The developmental progression of *skn-1a* mutants is identical to the wild type. The developmental progression of *eel-1* mutants is delayed, and this effect is exacerbated in *eel-1 skn-1a* double mutants. Error bars show mean ± SD. n = 20 animals per genotype. Ns p > 0.05; **** p < 0.0001, ordinary one-way ANOVA with Šídák’s multiple comparisons test. C) Images showing the effect of *skn-1a* and *eel-1* mutations on developmental progression. Animals were synchronized at the L1 stage by hatching in buffer overnight and then cultured in the presence of OP50 bacteria for 48 hours at 25ºC. Scale bar 300 μM. D) Quantification of animal length from (C). The developmental progression of *skn-1a* mutants is identical to the wild type. The developmental progression of *eel-1* mutants is delayed, and this effect is exacerbated in *eel-1 skn-1a* double mutants. Error bar shows mean ± SD. n > 13 animals per genotype. Ns p > 0.05; **** p < 0.0001, ordinary one-way ANOVA with Šídák’s multiple comparisons test.
(TIF)

S4 Fig Loss of EEL-1 does not cause accumulation of ubiquitin conjugates. Western blot for Ub conjugates. For each sample, lysates were prepared from 50 (day 1 adult) animals. *skn-1a* indicates *skn-1a*[*G2STOP*] and *eel-1* indicates *eel-1*[*G172STOP*]). Ubiquitin conjugates are increased in *skn-1a* mutants, but are not altered in *eel-1* mutants, and are not further increased in *eel-1 skn-1a* double mutants.
(TIF)

S5 Fig Sequence conservation of the EEL-1/HUWE1/Tom1 3xUBM domain. Multiple sequence alignment of EEL-1/HUWE1/Tom1 3xUBM domain. The invariant LP motif found in each UBM is in bold. Locations of amino acid substitution mutations analyzed in this study are indicated by red arrows.
(TIF)

S6 Fig *eel-1*[P3059A] mutation does not disrupt UbV-GFP degradation in the *skn-1a* mutant background. A) Fluorescence micrographs showing that accumulation of UbV-GFP is not increased by the *eel-1*[*P3059A*] mutation in the *skn-1a* mutant background. Scale bar 100 μM. B) Quantification of UbV-GFP levels shown in (A). All animals are *skn-1a* mutants. The x-axis label indicates the *eel-1* genotype. Error bars show mean ± SD. Results are shown for n = 15 animals. Ns p > 0.05, Welch’s t test.
(TIF)

S7 Fig Sequence conservation of the EEL-1/HUWE1/Tom1 UBA domain. A) Multiple sequence alignment of EEL-1/HUWE1/Tom1 UBA domain. The conserved L/MGF motif is in bold. L1446 is indicated by a red arrow. B) Alpha Fold 3 model showing predicted interaction between ubiquitin and the EEL-1 UBA domain. L1446 and hydrophobic residues of ubiquitin involved in the ubiquitin:UBA interaction are labelled.
(TIF)

S8 Fig Amino acid composition of EEL-1 and orthologs showing conserved lysine deficient regions. For each protein, red lines indicate locations of lysine residues. The location of the Acid-IDR domain (blue) and 3xUBM domain (black) within each ortholog is indicated. The location of the Acid-IDR was inferred by analysis of each ortholog’s sequence composition (see S9 Fig).
(TIF)

S9 Fig Amino acid composition of EEL-1 and orthologs showing conserved regions enriched in acidic amino acids. For each protein, the percentage of acidic (glutamate or aspartate) residues within a moving 100 amino acid window is plotted. Grey lines indicate lysine-deficient regions of each ortholog (see S8 Fig).
(TIF)

S10 Fig Vulval rupture of *eel-1*[*E2412K*] and *eel-1*[*E3064K*] mutants. A) Quantification of vulval rupture in day 2 adults. Results are shown for n = 3 replicate experiments; 25–35 animals were assayed in each replicate. Error bars show mean ± SD. Ns p > 0.05, **** p < 0.0001, indicates P-value compared to the wild type (WT) control, ordinary one-way ANOVA with Šídák’s multiple comparisons test. B) Quantification of vulval rupture in day 4 adults. Results are shown for n = 3 replicate experiments; 25–35 animals were assayed in each replicate. Error bars show mean ± SD. Ns p > 0.05, **** p < 0.0001, indicates P-value compared to the wild type (WT) control, ordinary one-way ANOVA with Šídák’s multiple comparisons test.
(TIF)

S11 Fig The HA::EEL-1 tag does not disrupt function. A) Fluorescence micrographs showing accumulation of UbV-GFP in *skn-1a* and *skn-1a*; HA::*eel-1* animals. All images are of L4 stage animals. Scale bar 100 μM. B) Quantification of UbV-GFP levels shown in (A). Error bars show mean ± SD. n = 45 animals per genotype. ns p > 0.05, Welch’s t test.
(TIF)

S1 Table Vulval rupture statistics. (XLSX)

S2 Table *C. elegans* strains used in this study. (XLSX)

S3 Table Sequence information of all guide RNAs and homology-directed repair (HDR) oligos used for gene editing in this study. (XLSX)

S1 Data Numerical data for all graphs. (XLSX)

S1 Raw Gel Raw Gel. Raw un-cropped images for all gels.
(PDF)
