# Supplementary material for: Lysine deficiency within a conserved lysine desert is critical for EEL-1/HUWE1 to support ubiquitin proteasome system function
Source: PLoS Genet. 2026 Aug 10;22(8):e1012273. doi: 10.1371/journal.pgen.1012273 (PMC13472506; doi:10.1371/journal.pgen.1012273)

uncropped gel images from Figure 7

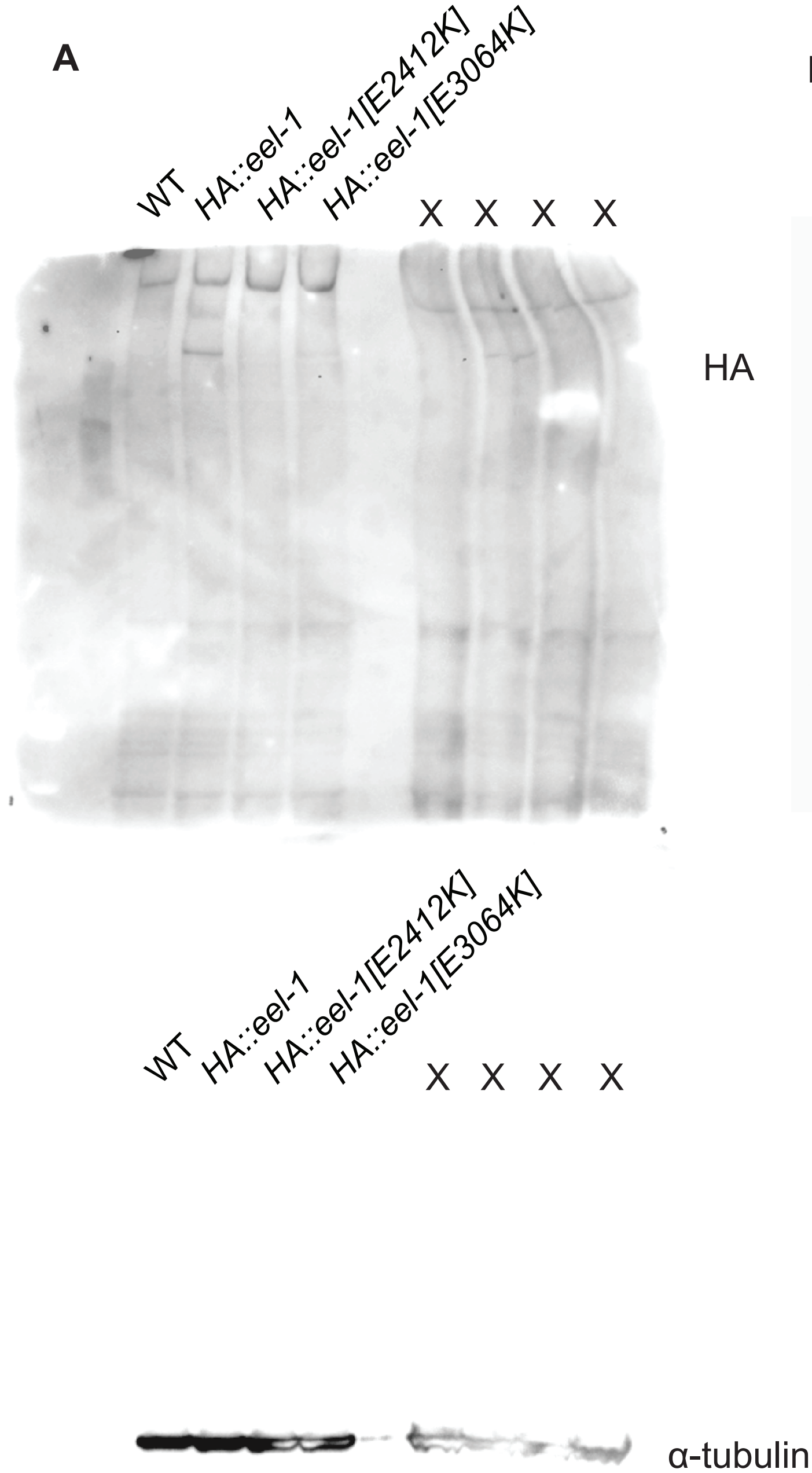

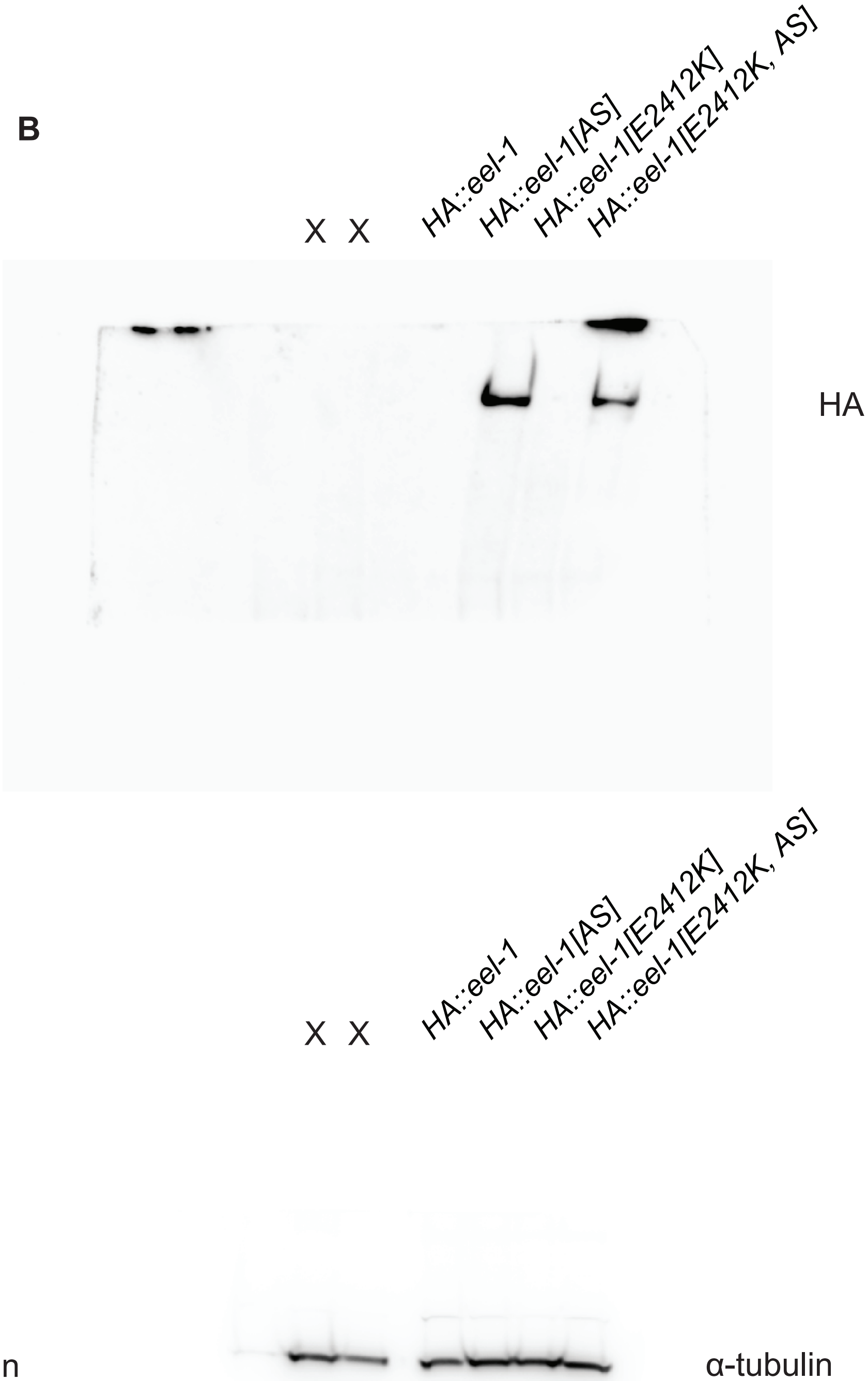

uncropped gel images from Figure S4

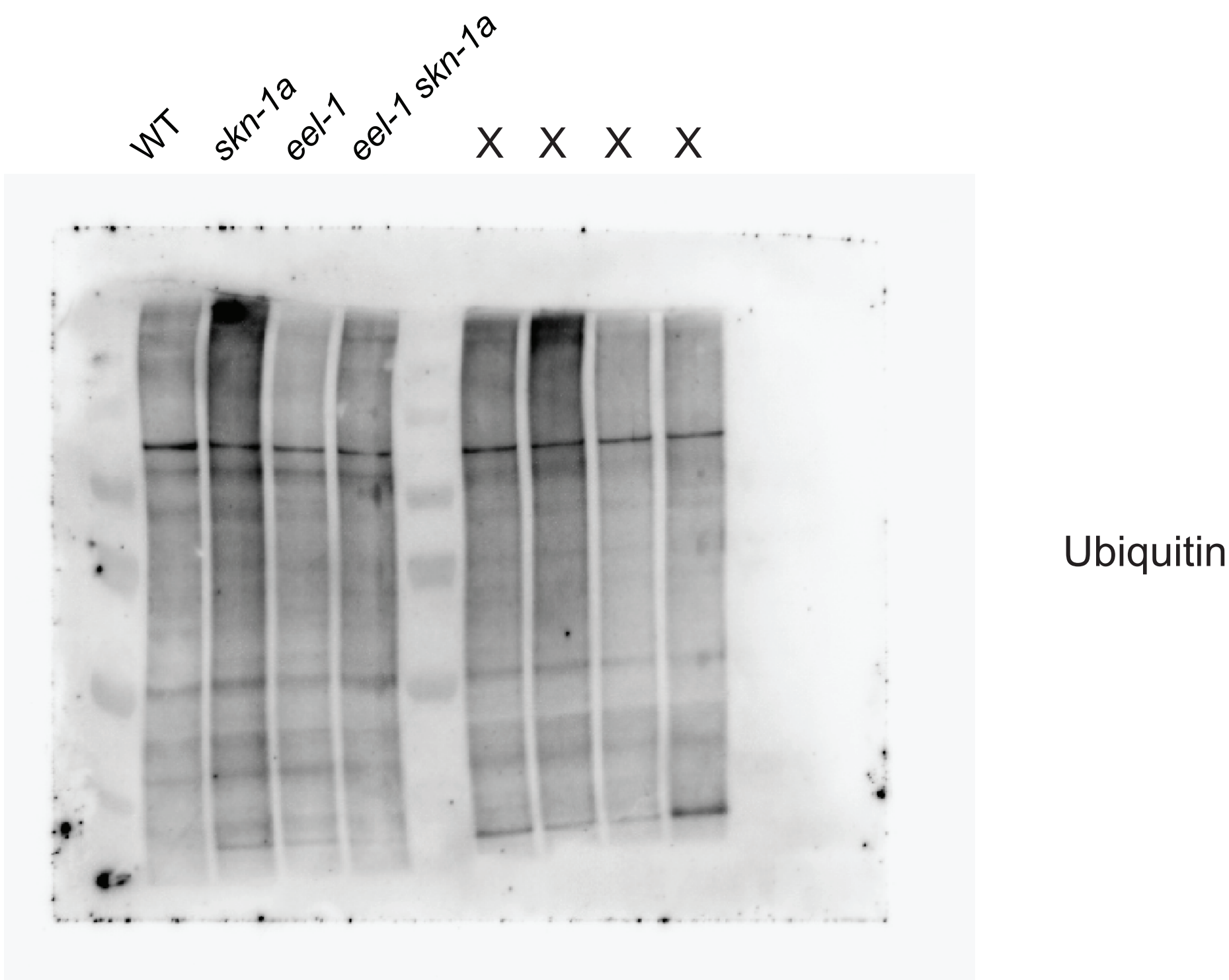

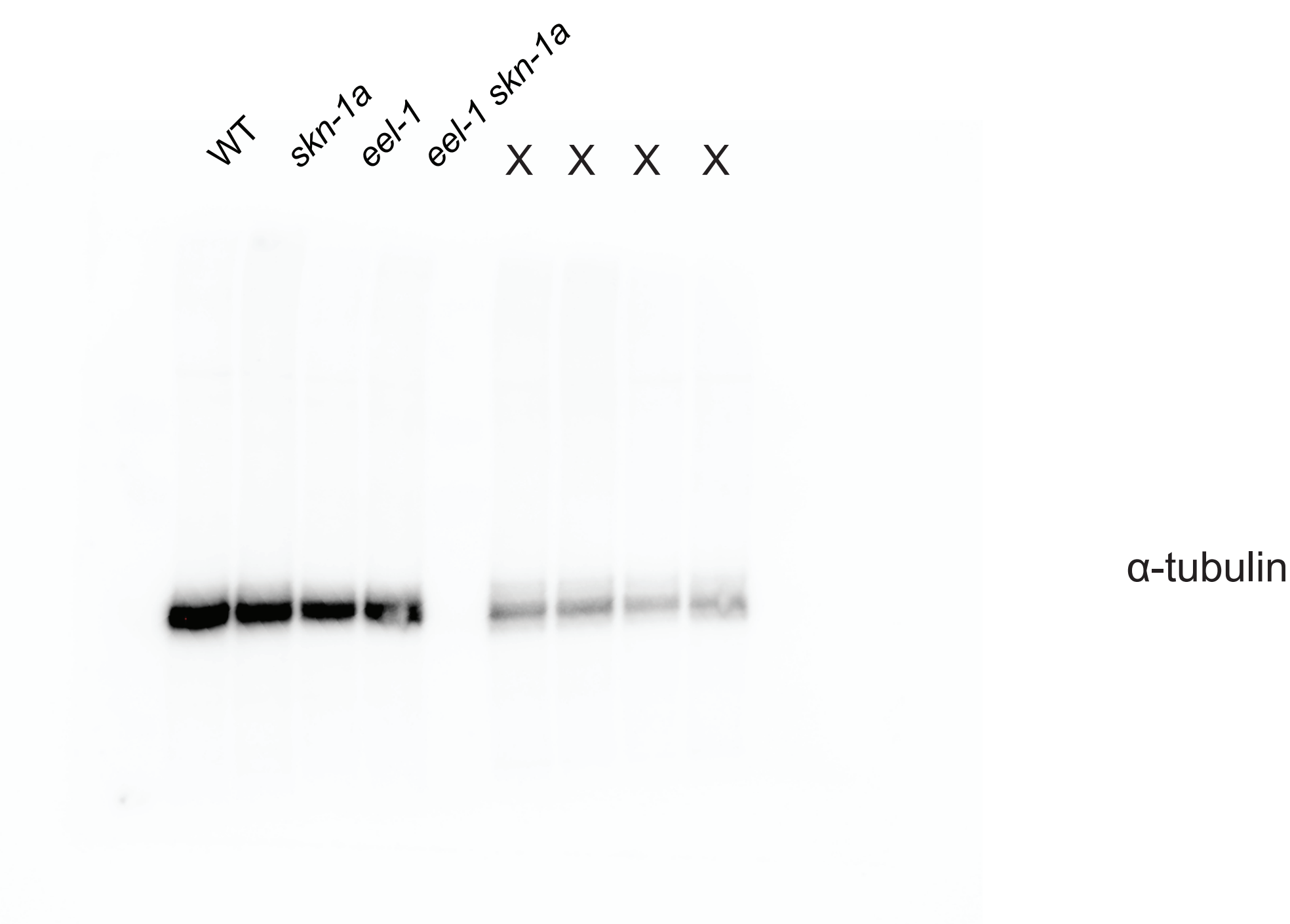

Supplement: Supplementary file 1 [file pgen.1012273.s016.pdf]
